# Virus-host protein-protein interactions of mycobacteriophage Giles

**DOI:** 10.1038/s41598-017-16303-7

**Published:** 2017-11-28

**Authors:** Jitender Mehla, Rebekah M. Dedrick, J. Harry Caufield, Jeroen Wagemans, Neha Sakhawalkar, Allison Johnson, Graham F. Hatfull, Peter Uetz

**Affiliations:** 1 Center for the Study of Biological Complexity, Virginia Commonwealth University, Richmond, VA 23284 USA; 2 Department of Biological Sciences, University of Pittsburgh, Pittsburgh, Pennsylvania 15260 USA; 3 Laboratory of Gene Technology, KU Leuven, Kasteelpark Arenberg 21 – Box 2462, 3001 Leuven, Belgium

## Abstract

Mycobacteriophage are viruses that infect mycobacteria. More than 1,400 mycobacteriophage genomes have been sequenced, coding for over one hundred thousand proteins of unknown functions. Here we investigate mycobacteriophage Giles-host protein-protein interactions (PPIs) using yeast two-hybrid screening (Y2H). A total of 25 reproducible PPIs were found for a selected set of 10 Giles proteins, including a putative virion assembly protein (gp17), the phage integrase (gp29), the endolysin (gp31), the phage repressor (gp47), and six proteins of unknown function (gp34, gp35, gp54, gp56, gp64, and gp65). We note that overexpression of the proteins is toxic to *M. smegmatis*, although whether this toxicity and the associated changes in cellular morphology are related to the putative interactions revealed in the Y2H screen is unclear.

## Introduction

Bacteriophages are the most abundant, diverse and highly populated biological entities with an estimated 10^31^ phage particles in the biosphere^1^. Mycobacteriophages are viruses of mycobacterial hosts, including *Mycobacterium smegmatis* and *Mycobacterium tuberculosis*, the causative agent of tuberculosis^2^. More than 1,400 completely sequenced mycobacteriophage genomes have been described (http://phagesdb.org)^3^, which not only have facilitated development of tools for mycobacterial genetics, but also may have therapeutic potential^4^. However, these genomes display high genetic diversity and encode an abundance of genes of unknown function^5,6^.

Determining the functions of phage genes will elucidate their mechanism of infection^7^. Efficient phage DNA replication is metabolically demanding, and phages often reprogram host nucleotide metabolism to their own benefit^8^. Transcriptomics and metabolomics studies in cyanobacteria show how phage can reroute the host metabolism, such as towards de novo fatty-acid synthesis, or to generate conditions suitable for virus assembly^9^. The increased rate of fatty acid biosynthesis, including triacylglycerol (TAG), may be a common strategy of viruses: lipid droplets/bodies that mainly contain TAGs serve as a source of energy during phage assembly^10^. This lipid remodeling may be an evolutionarily conserved strategy used by viruses to hijack host cell machinery. Also, phages are known to exploit other host biological processes including stress response and host replication^11^.

Many phage proteins regulate the host cell machinery by protein-protein interactions (PPIs) to propagate their progeny^12,13^. This offers an advantage to the phage by facilitating the production of suitable conditions for phage propagation^14^. For example, viruses can modulate the host glycome either by regulating host glycosyltransferases or by producing their own glycosyltransferases. The virus-encoded glycosyltransferases are predicted to be involved in a variety of virus–host interactions^15^. Other phage-encoded homologues of host proteins have been shown to act as endonucleases, sigma factors, RNases or heat-shock proteins^16–18^. However, only few phages have been systematically studied for molecular interactions between phage and host proteins^19^.

Mycobacteriophage Giles is a temperate phage that forms stable lysogens in *M. smegmatis* ^20^. It has a 53,746 bp genome and contains 78 putative protein-coding genes. The repressor (gene 47) is positioned approximately 65% of the genome length from the left genome end, and genes to its left include the virion structure and assembly gene, the integration cassette, and the lysis genes. Many of the genes to its right are of unknown function, but include those coding for a recombination system (RecET-like), DnaQ, DNA Methylase, RuvC, and WhiB^20^. Transcriptomic studies show that these genes to the right of the repressor are expressed early in lytic growth, and those in the left part of the genomes are expressed late in lytic growth^20^. A broad search to define Giles genes needed for lytic growth showed that more than half of the non-structural genes are dispensable for plaque formation, although many show minor defects in phage production^20^. These genetic and transcriptomic analysis support further analysis of mycobacteriophage Giles to elucidate protein-protein interactions^21^.

Here, we implemented a search for Giles-encoded proteins that interact with host proteins and may play roles in reprogramming of the host machinery. Ten Giles proteins were screened against host proteins using a yeast two-hybrid (Y2H) approach, and a network of 25 interactions was identified. Several of these interactions were pursued with phenotypic screens.

## Results

### Selection of Giles proteins

**Figure 1:**
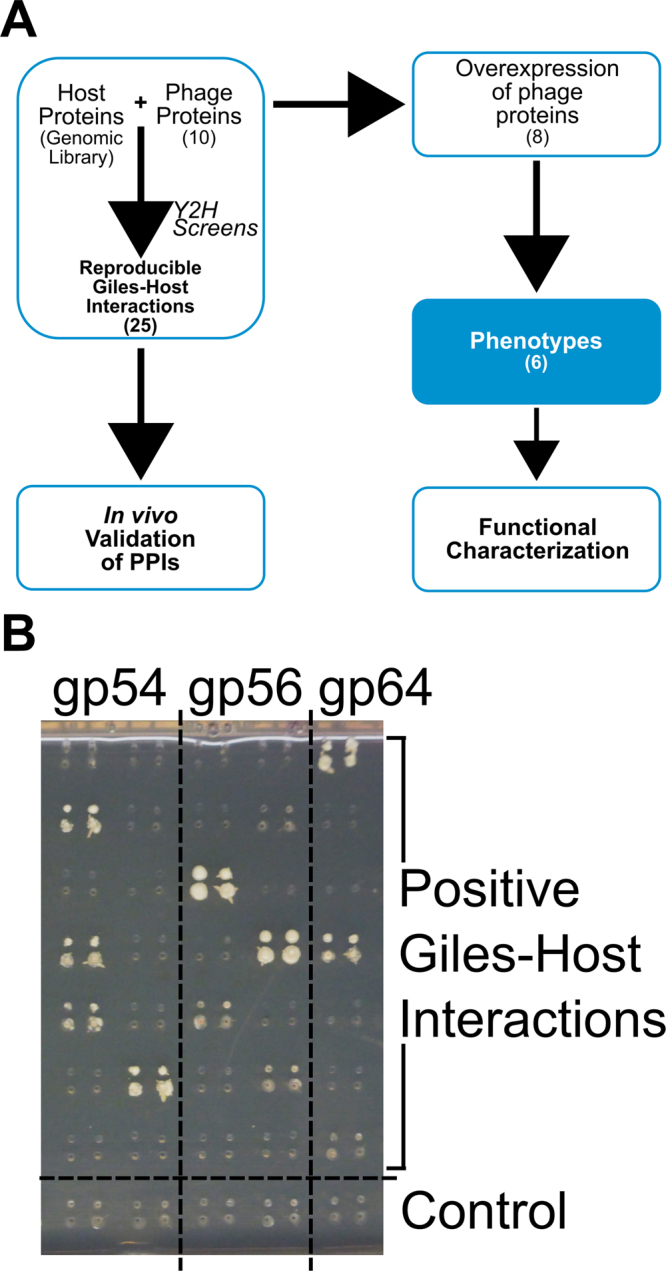
Y2H screens of *M. smegmatis* proteins with mycobacteriophage Giles proteins. **(A)** An overview of the methods used in this paper. **(B)** A representative array-based Y2H screen showing positive interactions for three Giles phage bait proteins. The baits were tested against host prey proteins and a control (empty prey vector). See methods for technical details.

A set of 10 Giles-encoded proteins (Table 1) were selected for screening against a *M. smegmatis* genomic library in a Y2H screen (Fig. 1A). These proteins represent a variety of expression and functional features, and include a putative virion assembly protein (gp17), the phage integrase (gp29), the endolysin (gp31), the phage repressor (gp47), and six proteins of unknown function (gp34, gp35, gp54, gp56, gp64, and gp65). Prior studies have shown that genes encoding the integrase (29) and repressor (47), together with genes 54 and 56 are not required for lytic growth^22^. In contrast, gene 64 is essential and is implicated in phage DNA replication^22^. The endolysin is required for lytic growth and the inability to delete genes 34, 35, and 65 suggests that these may also be required^22^. All ten proteins have few known interactions with other Giles-encoded proteins^21^, hence we speculated that they are likely to interact with the host.

**Table 1:** Protein-protein interactions (PPIs) of mycobacteriophage Giles and host *Mycobacterium smegmatis*. 3-ATS = 3-AT score^21^; *AMG = phage-encoded auxiliary metabolic genes^34^.

Mycobacteriophage Giles		*Mycobacterium smegmatis*								
Bait	Predicted function	Gene ID	Host protein	Interaction domain (Protein fragment encoded by clone sequence)	Size of Interaction domain	% of full length protein	Protein size (amino. acids)	3-ATS	*Putative auxiliary metabolic gene (Function of Host proteins in viral/phage metagenome)	Host protein domain
gp17	Tail chaperone	MSMEG_1662	taurine-pyruvate aminotransferase /Aminotransferase class III	138–365	227	49.24	461	0	other (class I & II aminotransferase are AMGs)	AAT_I superfamily (catalytic residues)
gp17		MSMEG_5776	PhoU family transcriptional regulator	18–130	112	50.45	222	50	Class II AMG	PhoU domain
gp29	Integrase	MSMEG_2001	conserved hypothetical protein/sugar transporter	142–219	77	35	220	66.66	other	
gp29		MSMEG_1438	50 S ribosomal protein L450S ribosomal protein L23	first 85	85	85	100	0	transcription, translation, protein synthesis	
gp31	LysinA	MSMEG_5574	sugar ABC transporter substrate-binding protein (PBP_Type 2 superfamily)	64–181	117	26.17	447	66.66	transport	
gp31		MSMEG_2071	TetR family transcriptional regulator	25–182	157	77.33	203	20	transcription, translation, protein synthesis	HTH motif DNA binding
gp34	Unknown	MSMEG_4441	Cupin domain protein	126–252	126	41.72	302	98	other	Interact with non-CUPIN_2 domain
gp35	Unknown	MSMEG_1130	hypothetical protein	55–154	99	26.4	375	66.66		
gp47	Putative Repressor	MSMEG_5625	Cyclododecanone Monoxygenase	243–375	132	21.56	612	50	AMG (but ammonia/methane monooxygenase)	AANH_Like superfamily
gp47		MSMEG_1245	phosphoadenosine phosphosulfate reductase	42–232	190	67.37	282	50		AANH_Like superfamily/PAPS reductase (active sites)
gp47		MSMEG_3811	universal stress protein	1–101	100	68.02	147	50	other	USP domain
gp47		MSMEG_1272	Putative ribosylglycohydrolase	1–132	131	26.62	492	50	other	ADP_Ribosyl_GH
gp54	Unknown	MSMEG_2161	FADD9 protein	55–240	185	71.98	257	90		
gp54		MSMEG_4430	ATP-dependent transcriptional regulator, MalT-like LuxR family	567–759	192	25.29	759	90		
gp54		MSMEG_6459	ferredoxin-dependent glutamate synthase 1	219–346	127	8.23	1542	20		Glutamine amidotransferase type-2
gp56	Unknown	MSMEG_4731	acyl-CoA synthetase/Fatty-acid-CoA ligase FadD23	44–253	209	36.22	577	98		AMP_binding
gp64	Unknown	MSMEG_3483	MOSC domain protein/Pyr kinase domain	1–194	193	87.33	221	66.66	other	
gp64		MSMEG_6699	conserved hypothetical	9–118	109	37.71	289	66.66		
gp64		MSMEG_3746	CTP synthetase	50–200	150	25.42	590	66.66		Amidoligase domain
gp64		MSMEG_5478	hydroxypyruvate isomerase	64–239	175	63.17	277	50		AP_endonuc_2
gp64		MSMEG_4154	transposase, Mutator family protein	12–282	270	69.05	391	0	other	
gp64		MSMEG_2367	glutamyl-tRNA amidotransferase	37–88	51	10.13	503	0		
gp65	Unknown	MSMEG_1954	ABC1 family protein/ubiB domain	267–417	150	34.16	439	66.66		
gp65		MSMEG_6702	[NADP + ] succinate-semialdehyde dehydrogenase	63–265	202	43.91	460	66.66	Class1 AMG	succinate-semialdehyde dehydrogenase 1-like
gp65		MSMEG_5641	glycosyl transferase, group 1 family protein (GTB_type superfamily)	88–125	37	9.66	383	0	AMG	(33–148) Glyco_trans_4-like_N, and (190–328) Glycos_transf_1

### Library screens detect interactors for Giles proteins

**Figure 2:**
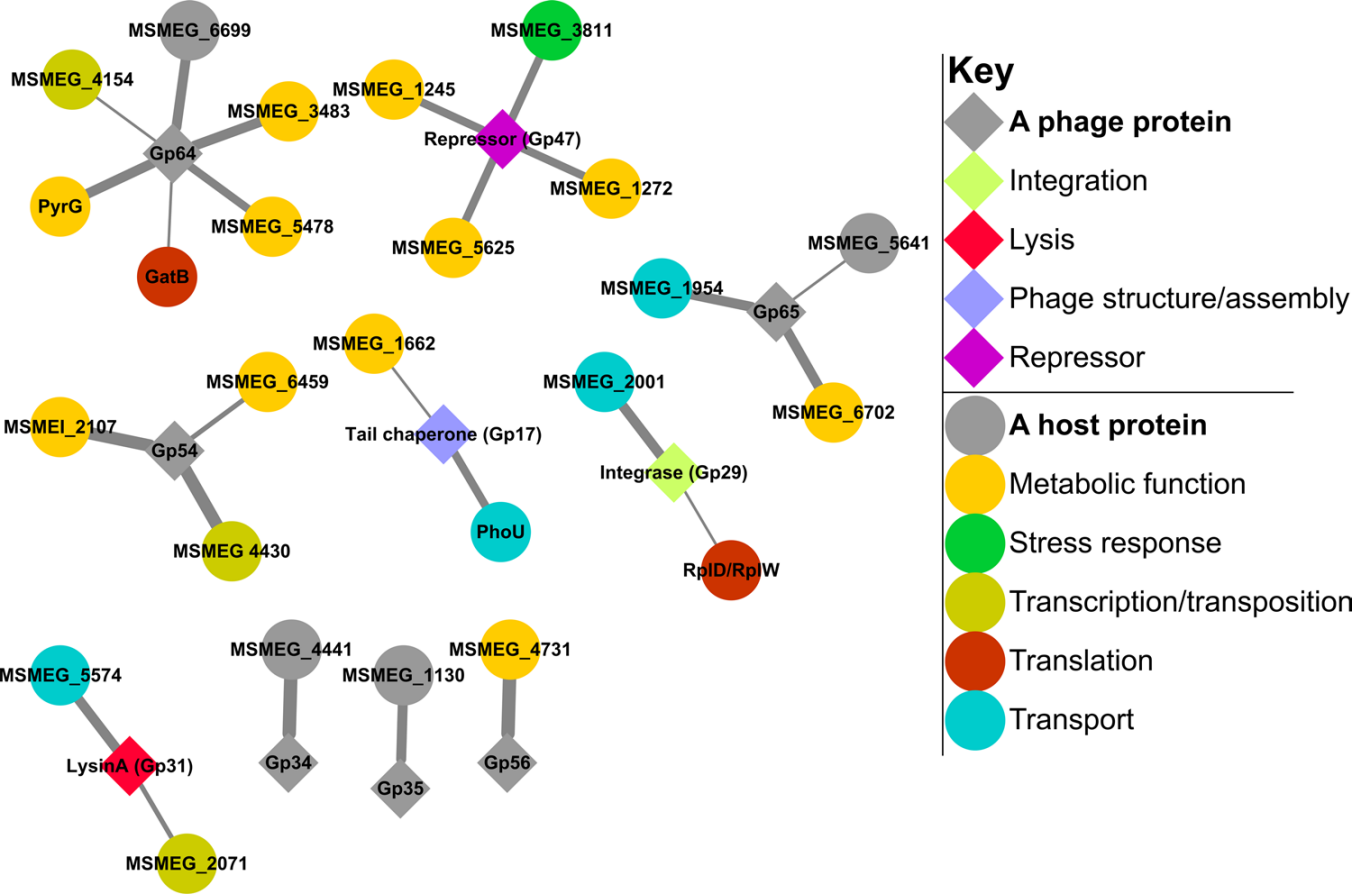
The Y2H *M. smegmatis*-Giles PPI network. Only reproducible PPIs are shown here. The line width corresponds to interaction strength for each interacting pair (as measured by 3-AT concentration, see methods).

Baits were screened against a custom-made *M. smegmatis* genomic library as described in the Methods. Approximately eight positive clones for each bait (Giles protein) were selected and sequenced to identify interacting prey partners from the host, except for baits gp54, gp56 and gp64, for which 12–16 clones were sequenced each. Positive clones were then retested using an array-based Y2H screen (Fig. 1B **)**, and a total of 78 positive clones were sequenced. After removing redundant sequences, we identified 59 Giles-host PPIs and these were retested in an independent Y2H experiment using freshly prepared clones for the host prey proteins (Fig. 1B); the sequences of the host proteins (interactors) are shown in Table S1. The reproducible interactions from the re-test screens were used to construct a Giles-host network of 25 reproducible interactions (Fig. 2).

### Size and domains of interacting fragments

**Figure 3:**
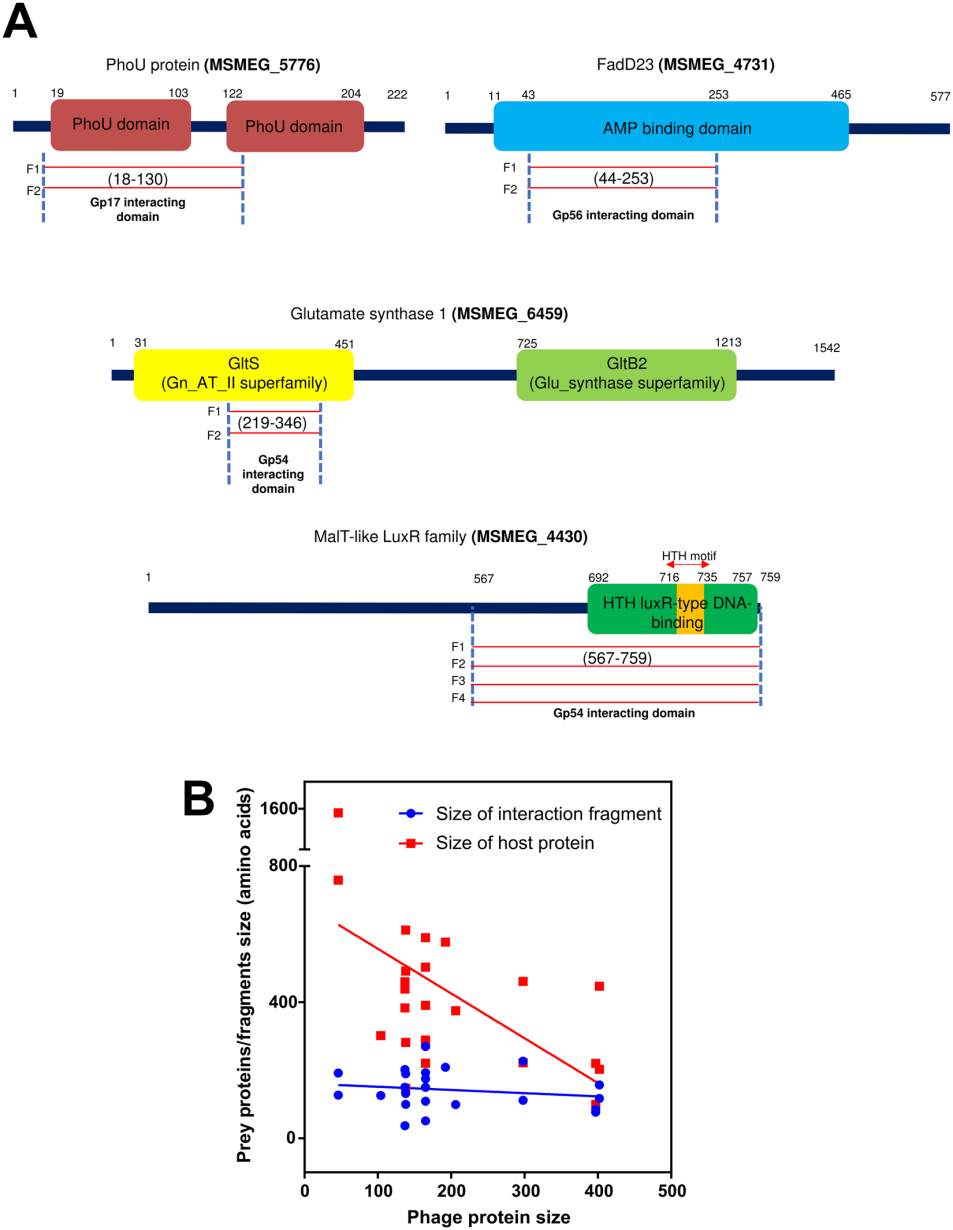
Mapping interacting domains of host proteins. **(A)** Giles Gp17 appears to interact with only one of the two PhoU domains of host PhoU (MSMEG_5776) protein. Similarly, Gp54 interact with the GltS domain of Glutamate synthase 1 (MSMEG_6459) and the HTH-DNA binding domain of MalT-like transcriptional regulator (MSMEG_4430). Also, Gp56 interact specifically with the AMP-binding domain of host FAD23 (MSMEG_4731) protein. Each red line indicates an independent clone (but identical), encoding the same region of the protein. Numbers (in brackets) denotes the size of interaction domain/fragment (start and end amino acids). **(B)** While the actual size of phage and host proteins vary widely, most interaction domains were in the range of 100–200 amino acid residues, as defined by the size-fractionated prey library (see methods).

For four of the putative Giles-host interactions, more than one independent interacting clone was isolated (two each for Giles gp17, gp56, and two and four for each of the two host proteins interacting with gp54, Fig. 3A). However, the host clones are identical and thus likely represent sibling clones in the library.

Multiple positive prey clones encoding the same fragments of interacting proteins were found for 3 Giles proteins (Gp17, Gp54 and Gp56). For example, Gp54 interacts with glutamate synthase 1 (MSMEG_6459) & MalT-like (Maltose-transcriptional) regulator (MSMEG_4430) and two and four fragments encoding the same protein region were found as interacting partners, respectively. These fragments helped to identify the interacting domain or region within the interactors. For example, only the GltS domain of glutamate synthase 1 (MSMEG_6459) was found to interact with Gp54 (Fig. 3A). However, the library nor the screens were saturated, so that additional or overlapping fragments may have been missed. The information of the interaction domain (or the protein fragment encoded by clone sequence) for all interactors, is shown in Table 1. Given that our prey library was size fractionated (see Methods), most PPIs domains were in the range of 100–200 amino acids (Fig. 3B).

### Phage proteins targeting similar processes in different hosts

Although the Giles-host protein interactions seem robust and reproducible in the Y2H screen, they could be spurious positive hits resulting from ‘sticky’ protein interactions, or they may reflect biologically relevant interactions involved in the growth of phage Giles. Because phage-host interactomes have been previously described for *Streptococcus* phages Cp1, Dp1 and *E. coli* phage lambda, we searched for interactions that are shared between these data sets (Table 2). In general, the interactomes are quite different to each other, likely reflecting the genetic diversity of these phages. However, phage proteins attacking the same host pathway or protein may reflect shared infection strategies. For instance, we note that both Giles gp17 and Dp-1 gp9 appear to interact with the host PhoU protein^23^. Whether this interaction is relevant for the phage remains unclear though, given that Giles gp17 and Dp-1 gp9 are functionally unrelated (the function of Dp-1 gp9 is unknown; Giles gp17 is a putative tail assembly protein).

**Table 2:** Different phage proteins target similar host proteins. All Giles interactions are from this study.

Phage	Protein	host	Host protein
Giles	Gp17	*M. smegmatis*	PhoU (MSMEG_5776)
Dp-1	Gp9	*S. pneumoniae*	PhoU (SP_1395)^23^
Giles	Gp17	*M. smegmatis*	III taurine-pyruvate aminotransferase (MSMEG_1662)
Lambda	p45	*E. coli*	class I and class II aminotransferases (P39389)^35^
Giles	Gp47	*M. smegmatis*	PAPS reductase (MSMEG_1245)
Lambda	p37	*E. coli*	PAPS reductase (P17854)^35^
Giles	Gp54	*M. smegmatis*	glutamate synthase 1 (MSMEG_6459)
Cp-1	Gp10	*S. pneumoniae*	SP_1881, a glutamate racemase (P63640)^23^
Giles	Gp65	*M. smegmatis*	ABC1 family protein (MSMEG_1954)
Dp-1	Gp47	*S. pneumoniae*	ABC transporter (SP_0687)^23^
Giles	Gp65	*M. smegmatis*	succinate-semialdehyde dehydrogenase (MSMEG_6702)
Lambda	p80	*E. coli*	succinyl-CoA synthetase (P0A836)^35^
Giles	Gp65	*M. smegmatis*	glycosyltransferase protein (MSMEG_5641).
Dp-1	Gp58	*S. pneumoniae*	glycosyltransferase (SP_1606)^23^

### The Giles gp54 - MSMEG_4430 and gp64 - MSMEG_3746 interactions are not required for phage infections

**Figure 4:**
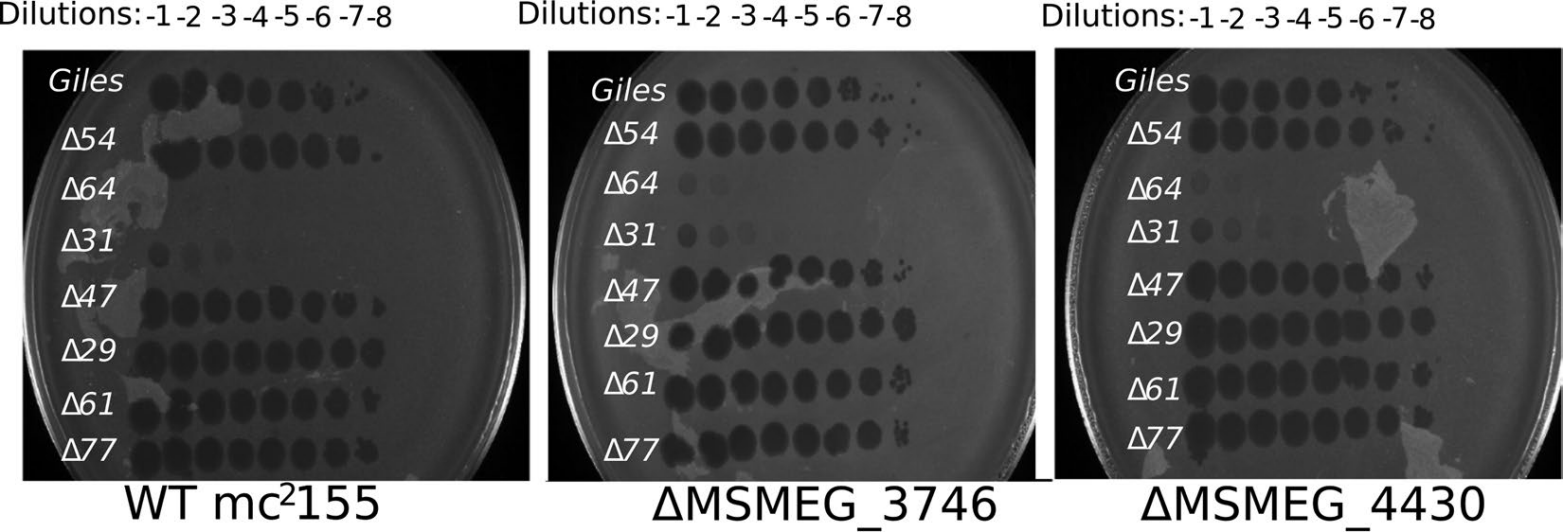
Phage infection assays did not validate the Giles-host PPI *in vivo*. Plating efficiency assays of gene deletion mutants of Giles (labeled on the left of each plate), which were plated on *M. smegmatis* deletions indicated at the bottom of each plate. There is no difference in plating efficiency with any of the Giles mutants when comparing each *M. smegmatis* mutant to WT mc^2^155.

To further explore the relevance of the Giles-host interactions, we determined whether Giles mutants with deletions of interacting non-essential genes had altered plating efficiencies on *M. smegmatis* mutants with deletions of host interaction protein genes (Fig. 4). However, no changes in plating efficiencies were observed, raising doubts as to whether these interactions are biologically relevant, although it remains plausible that some interactions are involved in roles that are not reflected in the plating assay.

### Phenotypes of Giles protein overexpression in bacteria

**Figure 5:**
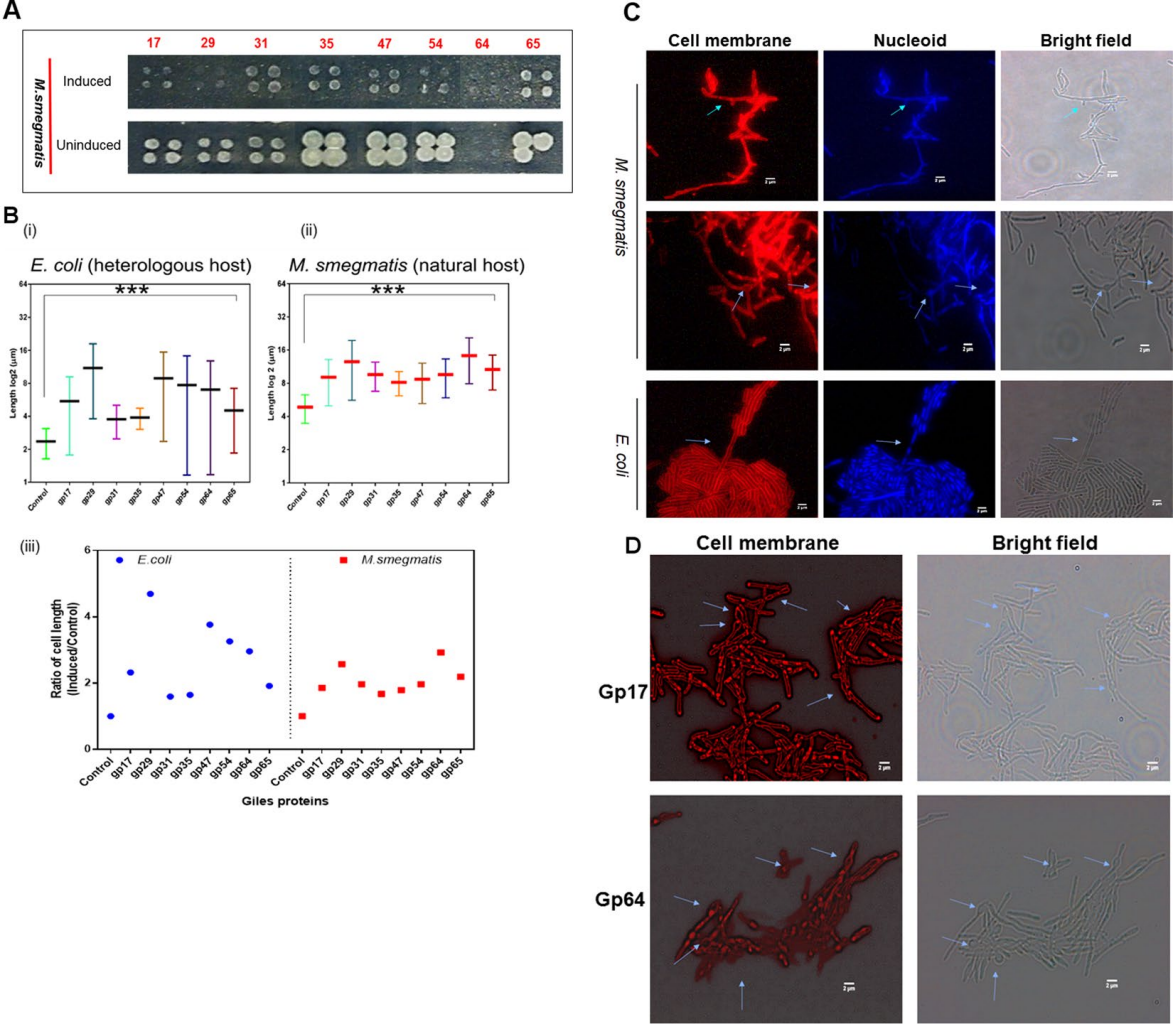
All overexpressed Giles proteins produce similar phenotypes. (**A)** Giles proteins were expressed in *M. smegmatis* on agar plates in the presence and absence of inducers (0.2 mM acetamide). Since all strains showed similar phenotypes, this is likely a nonspecific consequence of the overexpression. **(B)** The overexpression of Giles phage proteins increased the cell length in *E. coli* (i; approximate p-value < 0.0001 by the Kolmogorov-Smirnov test) and *M. smegmatis* (ii; approximate p-value < 0.0001 by the Kolmogorov-Smirnov test), suggesting that this is due to nonspecific consequences of the overexpression. The average fold difference in induced cells and control for *E. coli* and *M. smegmatis* are shown (iii). The controls were untransformed cells under similar conditions. **(C)** Overexpression of the Giles integrase (gp29) in *M. smegmatis*. The cells were stained with FM4–64 (Synapto Red C2) and DAPI to visualize cell membrane and nucleoid, respectively. Micrographs show *M. smegmatis* and *E. coli* cells 24 and 6 hours after induction, respectively. Arrows indicates the branching and the multiple nucleoids seen upon Giles protein expression. Scale bar represents 2 µm. **(D)** Morphological alterations in *M. smegmatis* upon Giles gp17 and gp64 expression. The micrographs were taken at 24 hours after induction. Arrows (in 5D) indicate the polar and septal bulging in *M. smegmatis*.

It has been shown previously that overexpression of phage proteins can be toxic to growth of the host, and for at least some of these that it is mediated by interactions with host proteins^13^. We therefore overexpressed eight of the Giles proteins in *M. smegmatis* and determined whether expression is inhibitory for growth, and if morphological changes occur in the cells. All eight genes inhibited *M. smegmatis* growth when overexpressed (Fig. 5A), but it is unclear whether the toxicity is a result of non-specific consequences of overexpression.

We observed that overexpression of all eight Giles proteins induced some cell lengthening, although no more than three-fold in the maximal effects (Fig. 5B). Because all of the genes behaved similarly, this is likely a non-specific result of general stresses placed on the cells under these conditions. Moreover, similar changes were observed when the same genes were expressed in *E. coli*, further supporting these as non-specific consequences of overexpression (Fig. 5C). We note that overexpression of Giles gp29 appears to induce some cell branches, perhaps as a consequence of DNA damage associated with non-specific DNA cleavage by the Giles integrase (Fig. 5C). Other cell deformations are observed when Giles gene 17, 64, and 54 are overexpressed including polar bulging and improper septal positioning (Fig. 5D, S3). We note that although such changes could be associated with inhibition of cell function mediated though the interactions identified in the Y2H screen, it remains possible that they reflect interactions between phage and host proteins that were not identified in the Y2H experiment.

## Discussion

Y2H data are often considered as unreliable, and fraught with a large number of false positives. The reasons for this are two-fold. First, many false positives can result from non-reproducible growth in the Y2H screen itself. Second, false positives can arise from proteins that are either ‘sticky’ or improperly folded. The first explanation seems unlikely for the Giles-host interactions described here, as the initial hits were extensively retested, and irreproducible positives eliminated. We can also rule out ‘stickiness’ as this typically results a large number of interactions which we did not find. Improper folding of the mycobacteriophage-encoded proteins in yeast may be more likely although we have no evidence that phage proteins are folding less well than others. We would argue that many interactions found in our study do in fact happen but may not be physiologically relevant. Given that irrelevant interactions are unlikely to have any negative impact on phage replication there should be little selective pressure to lose such interactions in phage evolution. Finally, it is also likely that some potential interactions were not identified, as false negatives also are common in Y2H screens^24,25^.

We note that for most of the proposed interactions, it is difficult to envisage what role they play in the biology of the phage. For example, the interaction between Giles gp17 and *M. smegmatis* PhoU (MSMEG_5776) is one of the strongest (as measured by a 3-amino-triazole (3-AT) concentration of 50 mM) and seemingly robust in the Y2H screen. However, Giles gp17 is a putative tail chaperone protein, and is unlikely to play any role in phosphate metabolism during infection.

One of the more plausible interactions we observed is between Giles gp64 and the host CTP synthase (MSMEG_3746). Deletion of Giles 64 results in a defect in phage DNA replication^22^, and because CTP synthase is involved with nucleotide metabolism, interactions between the two proteins would conceivably play a role in phage DNA replication or its control. We note, however, that MSMEG_3746 is not essential for *M. smegmatis* growth, and it is more likely that Giles gp64 plays a more direct role in phage DNA replication. Nonetheless, this interaction might be worthwhile examining further to determine if the two proteins interact biochemically.

In summary, we have described here an initial screen to identify phage Giles-encoded proteins that interact with *M. smegmatis* proteins. It is plausible that some of these are relevant to the growth of phage Giles, although our screen – like other Y2H screens – produced many positive clones that may be physiologically irrelevant and thus will require substantial further analysis to elucidate which are of greatest interest. The phenotypes resulting from overexpression of Giles phage proteins are consistent with at least some of these resulting from interactions of host proteins and inactivation of their function, but it remains to be seen if these are the same as those identified in the Y2H experiment, or whether they result from different interactions that are missed as false negatives in the Y2H screen.

## Materials and Methods

### Bacterial strains and growth conditions

*M. smegmatis* mc^2^ 4517 was cultured in Difco^TM^ Middlebrook 7H9 broth (BD) supplemented with ADC (5 g/L albumin, 2 g/L dextrose, 3 g/L catalase) and 0.05% Tween-80. Selection was performed on solid agar plates with 100 μg/ml hygromycin, 50 μg/ml kanamycin, for both liquid and solid media^26^. *E. coli* TOP10 and DH5α were used for cloning and were cultured in LB broth or agar. *E. coli* was selected at 150 μg/ml hygromycin, 35 μg/ml chloramphenicol, for both liquid and solid media. All strains were grown at 37 °C. For protein expression, *E. coli* BL21 cells were used. All the expression experiments were done at 30 °C unless otherwise mentioned. For vector details, see Mehla *et al*. 2015^27^.

### Construction of a random genomic Y2H prey library of *M. smegmatis*

A random genomic fragment library from *M. smegmatis* mc^2^155 was constructed as follows. First, genomic DNA was isolated from a stationary 1 L *M. smegmatis* culture grown in 7H9 medium using the Belisle (1998) protocol^28^. Subsequently, 100 µg DNA was partially digested with AluI, after which the 400–1500 bp fraction was extracted from a 1% DNA agarose gel. 200 ng blunt DNA fragments were then ligated into 0.5 µg pENTR 1 A (Thermo Scientific) (1:1 molar ratio), which was cut with DraI and EcoRV and dephosphorylated. The ligation mixture was transformed into electrocompetent *E. coli* MegaX DH10B cells (Thermo Scientific). Transformants (5.4 × 10^6^, 159 × redundancy of the 6.99 Mbp genome) were pooled and stored at −80 °C (20% glycerol). Next, pENTR 1 A/*M. smegmatis* library plasmid DNA was isolated from a 4 ml overnight culture. 500 ng was then subcloned using a Gateway LR reaction to 500 ng pGADT7g yeast two-hybrid prey vector, which was also transformed into electrocompetent *E. coli* MegaX DH10B cells. Again, 1.7 × 10^6^ transformants (at 8 × redundancy, taking into account the correct reading frame fusion between the Gal4p activation domain and the inserted coding sequence) were pooled and used for a random genomic fragment library prep (average length of 950 bp).

### Gateway cloning

The ORFs for the Giles proteins were cloned into the Gateway compatible Y2H vector pGBGT7g using LR reaction of Gateway cloning as per supplier’s instructions (Invitrogen). The ORFs encoding Giles proteins, cloned as baits into pGBGT7g, were transformed into Y2H strain AH109^29^. For protein expression in *E. coli* and *M. smegmatis*, the ORFs encoding Giles proteins were also cloned in expression specific shuttle vector pDESTsmg^26^ using Gateway LR reactions.

### Yeast Two-Hybrid Screening

To characterize Giles-host interactions, we screened 10 phage proteins against a random genomic library of *M. smegmatis*. We used a Y2H library screening approach followed by array-based Y2H screens to verify the interactions found in the library screen^30^. Thus, Giles-host protein interactions were detected using both library- and array-based screens. The background growth was suppressed by 3-AT (3-amino-triazole) in Y2H screens to minimize the rate of false positives. The 3-AT score was calculated for PPIs as described previously^21^.

### Genomic library screens

The constructed random genomic library **(see section above**) was transformed into Y2H mating-compatible yeast strain Y187 and screened against the selected Giles phage proteins^30^. The Giles phage proteins were selected based on their essentiality^22^ and the coverage in our recently published Giles interactome^21^. Interacting preys from positive clones from library screens were identified by colony PCR and sequencing. Sequencing was done using a single forward primer at Eurofins Genomics, Louisville KY. The sequences were then analyzed for prey identification using blastN against the *M. smegmatis* genome. Colonies with no sequence reads were removed at this step.

### Array-based Yeast Two-Hybrid

Once the prey proteins were identified from library screening, the plasmids for interacting prey clones were isolated from yeast cells. Briefly, the cells were treated with Zymolyase^R^-100T (Sunrise Science Products, Inc.) followed by a standard protocol for plasmid isolation as per the suppliers’ manual (Macherey-Nagel Inc.). The isolated prey proteins were then transformed back into Y2H compatible yeast strain Y187 as previously described^29^. Then, the interactions between Giles baits and identified host prey proteins were tested using array-based Y2H method as previously described^27^.

### *In vivo* validation of protein-protein interactions

To validate selected Giles-host interactions, *M. smegmatis* KOs were constructed. A PCR reaction using primers MSMEG_XXXX A and B were used to amplify the 5′ end of the gene of interest in *M. smegmatis* from mc^2^155 DNA. Next, primers C and D were used to amplify the 3’ end of the gene of interest in *M. smegmatis* from mc^2^155 DNA **(**Table S2 **)**. The PCR products of these two reactions were then used, along with a purified hygromycin resistance gene, in a PCR to amplify the 1.3 kb substrate with primers A and D. This substrate was then electroporated into recombineering mc^2^155 cells and recombinants were selected with hygromycin containing media. Colonies were verified by PCR. KO mutants were then plated in a top-agar overlay onto 7H10 hygromycin plates. Phages were diluted in phage buffer and spotted on the overlay. The Giles gene deletion KOs were constructed as reported previously^22^.

### Protein expression in *E. coli* and *Mycobacteria*

All the selected phage proteins were expressed both in *E. coli* and *M. smegmatis mc* ^2^4517 (kindly provided by Prof. Shaun Lott) using a Gateway compatible shuttle vector (pDESTsmg). The vector and methodology details are described elsewhere^26^. The electrocompetent *M. smegmatis* cells were prepared in the lab as described previously^31^.

Briefly, the expression constructs of Giles phage proteins were electroporated in the electrocompetent *M. smegmatis* cells using a BioRad Gene Pulser (R = 1000 Ω, Q = 25 μF and V = 2.5 kV). The cells were plated on 7H9 medium (supplemented with ADC = Albumin-Dextrose-Catalase); 100 µg/ml of hygromycin and 50 µg/ml of kanamycin/Tween). About 5–6 clones were tested for each phage protein for expression on hard agar. For induction in mycobacteria, acetamide (0.2 mM) was added to the growth media or to solid agar plates.

For expression in *E. coli* BL21(pLys), the cells were transformed with expression constructs encoding Giles proteins and protein expression was induced using IPTG (0.5 mM) at 30 °C. For *E. coli*, LB plates with ampicillin (100 µg/ml) and chloramphenicol (35 µg/ml) were used.

Thus, Giles proteins were expressed in *M. smegmatis* and in *E. coli* both on 7H9 and LB solid agar and broth, respectively.

### Light microscopy and image analysis

The cells were stained and imaged to visualize cell membrane and nucleoid using FM4–64 (Synapto Red C2, Biotium Inc.) and DAPI respectively. The cells were imaged on an Olympus BX41 microscope at 100x in a dark room. Images were captured with a microscope digital camera “AmScope MU1400”. The ImageJ software^32^ was used for measuring cells dimensions/length (National Institute of Health).

### Data Availability

The protein interactions from this publication have been submitted to the IMEx consortium (http://www.imexconsortium.org) through IntAct^33^ and assigned the identifier IM-26164.

## Electronic supplementary material

Supplementary Information

Table S1
