# Supplementary material for: Virus-host protein-protein interactions of mycobacteriophage Giles
Source: Sci Rep. 2017 Nov 28;7:16514. doi: 10.1038/s41598-017-16303-7 (PMC5705681; doi:10.1038/s41598-017-16303-7)
Supplement: Supplementary file 1 — Supplementary Information [file 41598_2017_16303_MOESM1_ESM.pdf]

Virus-host protein-protein interactions of mycobacteriophage Giles

Jitender Mehla*[1], Rebekah M. Dedrick[2], J. Harry Caufield[1], Jeroen Wagemans[3], Neha Sakhawalkar[1], Allison Johnson[1], Graham F. Hatfull[2], Peter Uetz[1]*

1 Center for the Study of Biological Complexity, Virginia Commonwealth University, Richmond, VA 23284, USA

2 Department of Biological Sciences, University of Pittsburgh, Pittsburgh, Pennsylvania 15260, USA

3 Laboratory of Gene Technology, KU Leuven, Kasteelpark Arenberg 21 – Box 2462, 3001 Leuven, Belgium

Supplementary Figures and Tables

Figures S1- S4 The phenotypic alterations of natural and heterologous hosts upon overexpression of Giles proteins. Giles Gp17 in *E. coli* **(Fig S1 A)** and *M. smegmatis* **(Fig S1 B; arrow indicates formation of small cells)**; Gp47 in *E. coli* **(Fig S2 A)** and *M. smegmatis* **(Fig S2 B); Gp**54 in *M. smegmatis* **(Fig S3)** and Gp64 in *E. coli* **(Fig S4 top)** and *M. smegmatis* (**Fig S4 bottom**).

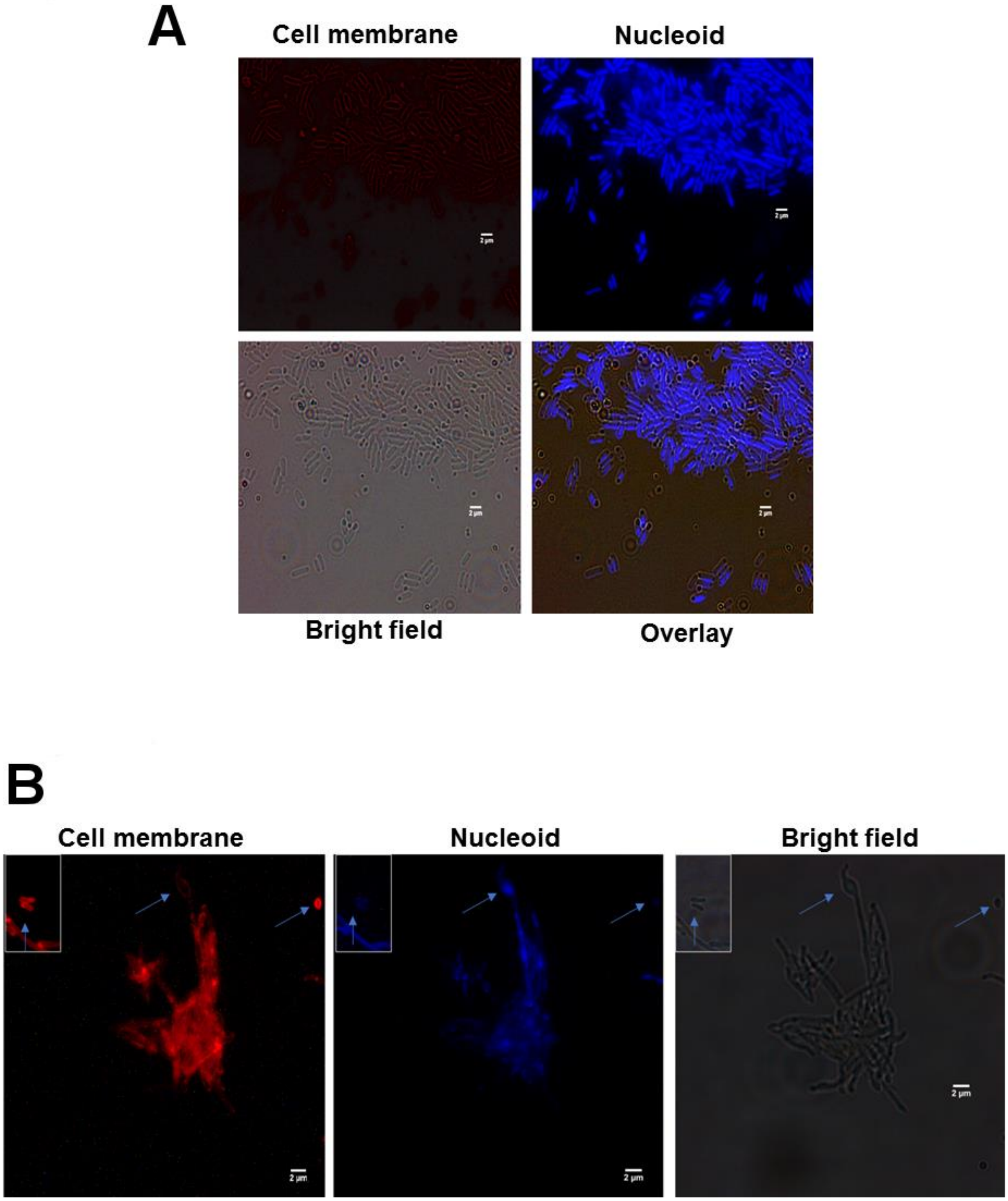

Supplementary Figure S1. The phenotypic alterations of natural and heterologous hosts upon overexpression of Giles proteins. Giles Gp17 in *E. coli* (**Fig S1 A**) and *M. smegmatis* (**Fig S1 B**; arrow indicates formation of small cells).

A

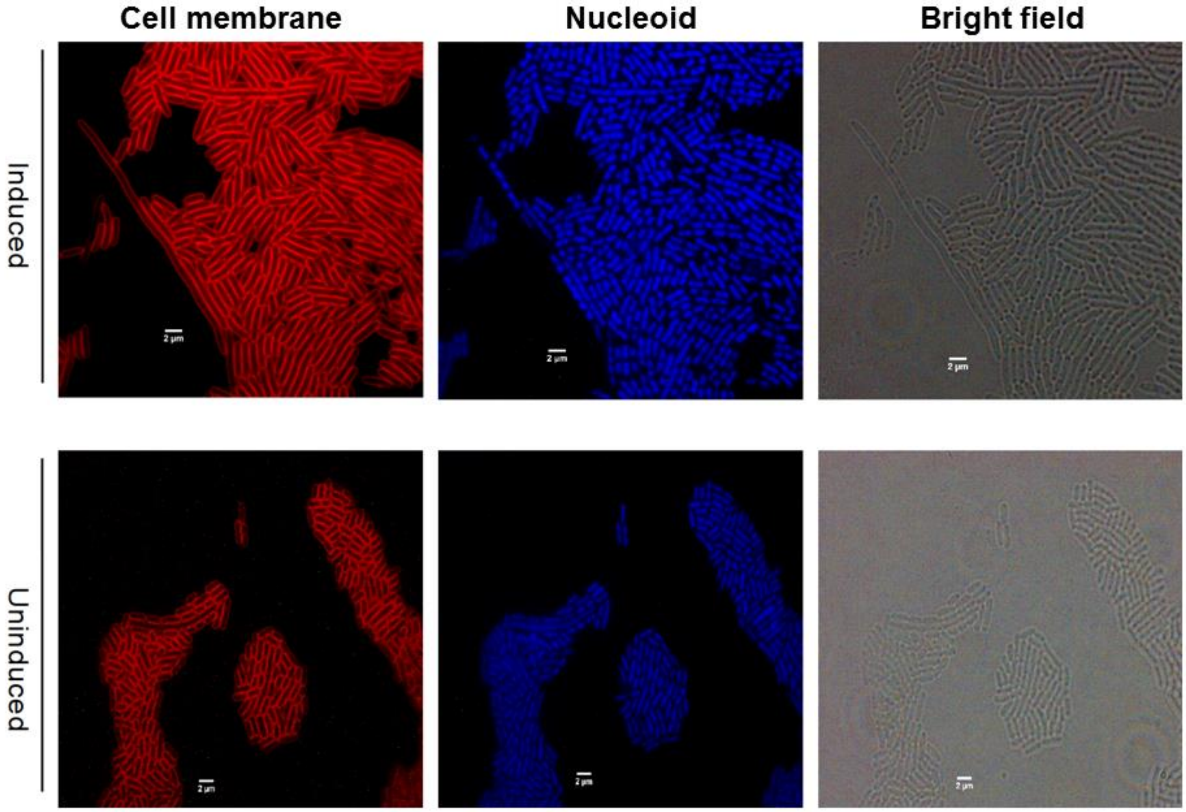

B

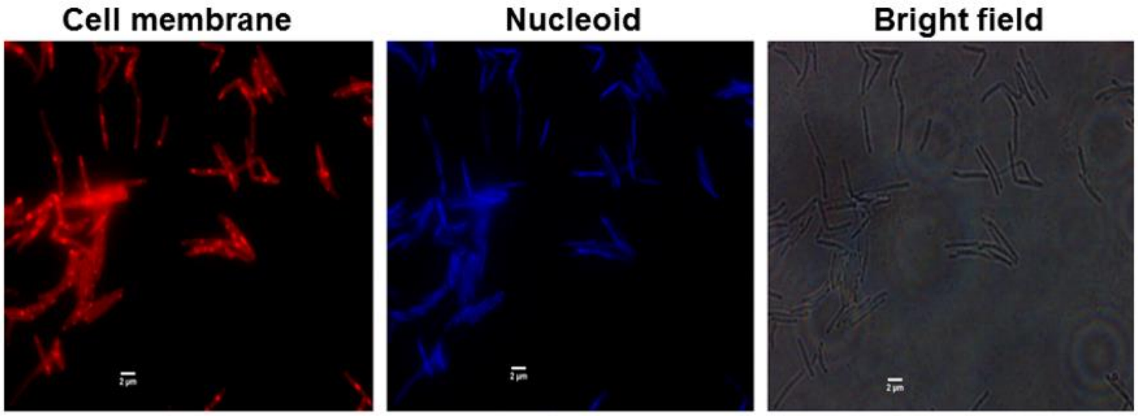

Supplementary Figure S2. The phenotypic alterations of natural and heterologous hosts upon overexpression of Giles proteins. Giles Gp47 in *E. coli* (**Fig S2 A**) and *M. smegmatis* (**Fig S2 B**).

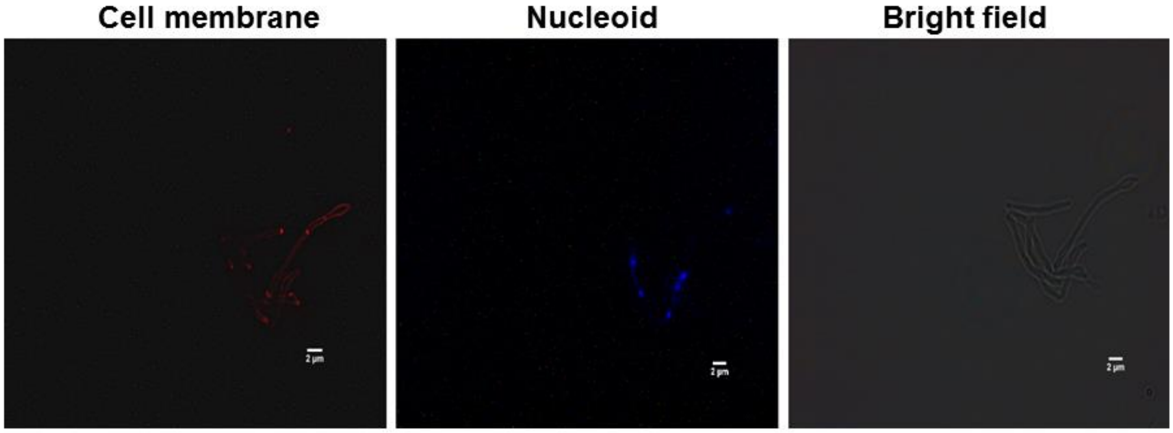

Supplementary Figure S3. The phenotypic alterations of *M. smegmatis* upon overexpression of Giles protein Gp54.

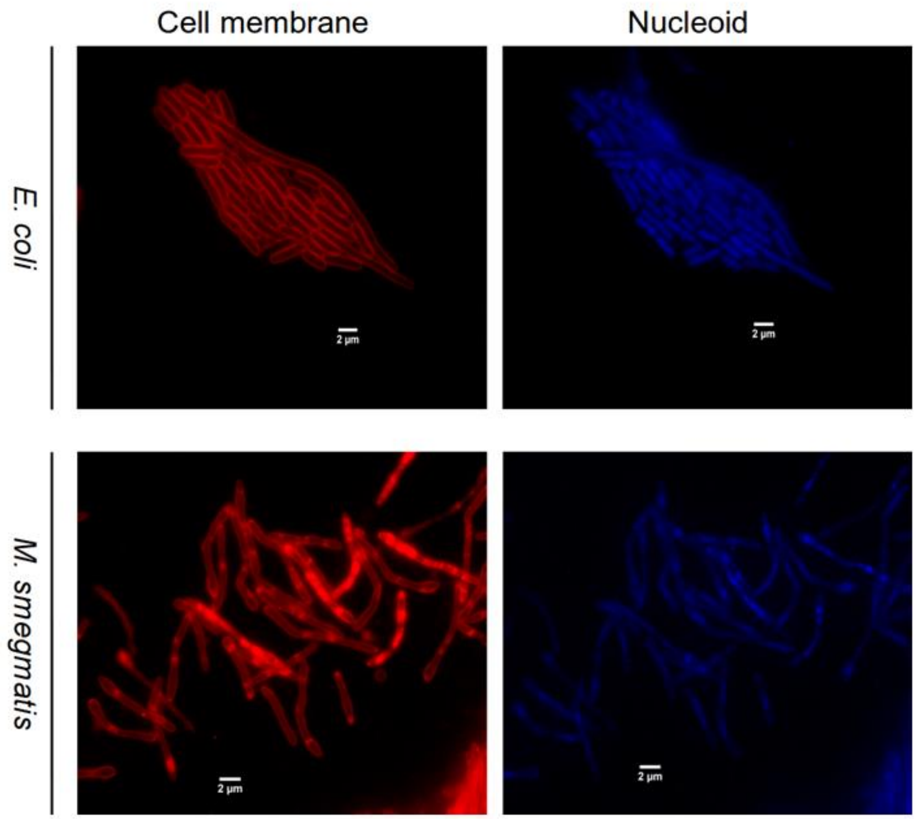

Supplementary Figure S4. The phenotypic alterations of natural and heterologous hosts upon overexpression of Giles proteins. Giles Gp64 in *E. coli* (**top**) and *M. smegmatis* (**bottom**).

Table S1. The sequences of the host interactors for Giles proteins with other details, are shown here. Separate Excel spreadsheet.

Table S2. List of oligonucleotides used in this study.

Oligo Name	Sequence
MSMEG_3746A	GCACGGATCGGGAACCGTCC
MSMEG_3746B	TGGTGAGGGAGATGAGGTCTGAAGAAGCATCCTCCGTGACGAAGGG
MSMEG_3746C	GTTGAGGTGTGAGGTGTGCTGAAGGCTGGAGAAATCCGACGTCCGTG
MSMEG_3746D	TGGCGGTATTGGTACACCAGTACCAC
MSMEG_4430A	CCAGCGATACAAAAGGTTTGAGGAAGATG
MSMEG_4430B	TGGTGAGGGAGATGAGGTCTGAAGGTCCGAACTCCACGAATTGAATGAATCC
MSMEG_4430C	GTTGAGGTGTGAGGTGTGCTGAAGGGTCGTGAACTCGGCTTGCTC
MSMEG_4430D	GGAGGATGTCCGATCGGTTGC
MSMEG_5773A	GTCGGCGAGGAACTGGGATGG
MSMEG_5773B	TGGTGAGGGAGATGAGGTCTGAAGGGCTTCTCCTGGTAGCTTCGGC
MSMEG_5773C	GTTGAGGTGTGAGGTGTGCTGAAGTCAAATCATTCTCCGGTGCACAGGAG
MSMEG_5773D	CGACGCCTACACCGTCACACC
